# Supplementary material for: Biodistribution of a Mucin 4-Selective Monoclonal Antibody: Defining a Potential Therapeutic Agent Against Pancreatic Cancer
Source: Int J Mol Sci. 2025 Jun 24;26(13):6042. doi: 10.3390/ijms26136042 (PMC12249516; doi:10.3390/ijms26136042)
Supplement: Supplementary file 1 [file ijms-26-06042-s001.zip › ijms-3616570-supplementary-xml.pdf]

Supporting Information

Biodistribution of a Mucin 4-Selective Monoclonal Antibody: Defining a Potential Therapeutic Agent Against Pancreatic Cancer

Achyut Dahal [1], Jerome Schlomer [2], Laura Bassel [2], Serguei Kozlov [2] and Joseph J. Barchi Jr. [1,*]

[1] Glycoconjugate and NMR Section, Chemical Biology Laboratory, The Center for Cancer Research, National Cancer Institute at Frederick, Frederick, MD 21702; barchij@mail.nih.gov

[2] Center for Advanced Preclinical Research; Frederick National Lab for Cancer Research, The Center for Cancer Research, National Cancer Institute at Frederick, Frederick, MD 21702

* Correspondence: barchij@mail.nih.gov; Tel.: +1-301-846-5905

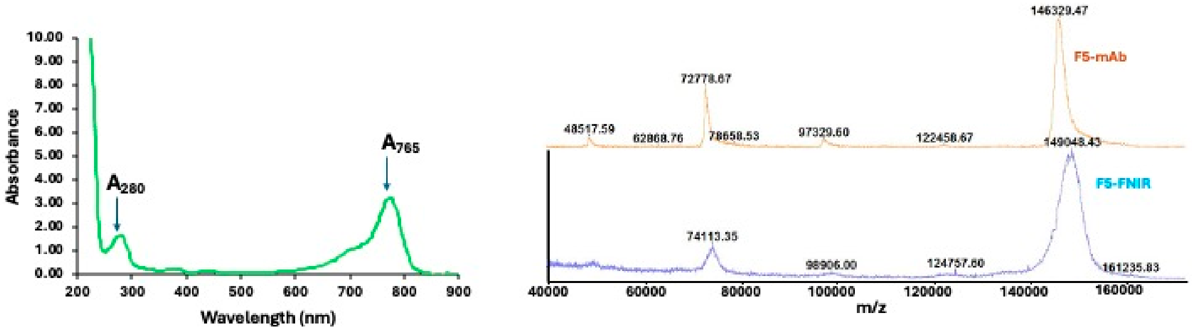

A_{280}	A_{765}	DOL calculated
1.6	3.03	**2.12**

F5 mab (Mw)	F5-FNIR (Mw)	DOL calculated
146329	149048	**2.35**

Figure S1. Comparison of UV Sspectroscopy (left) and MALDI Mass Spectrometry (right) calculations of the Degree of Labeling for the F5-FNIR conjugates

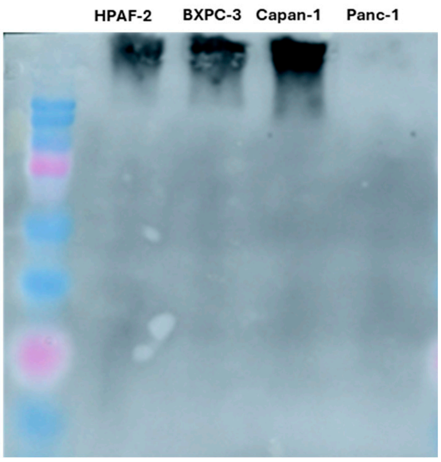

Figure S2. MUC4 expression validation by Western Blot of $MUC4^+$ CDX models whereas no the expression is observed in the PANC-1 $MUC4^-$ CDX model.

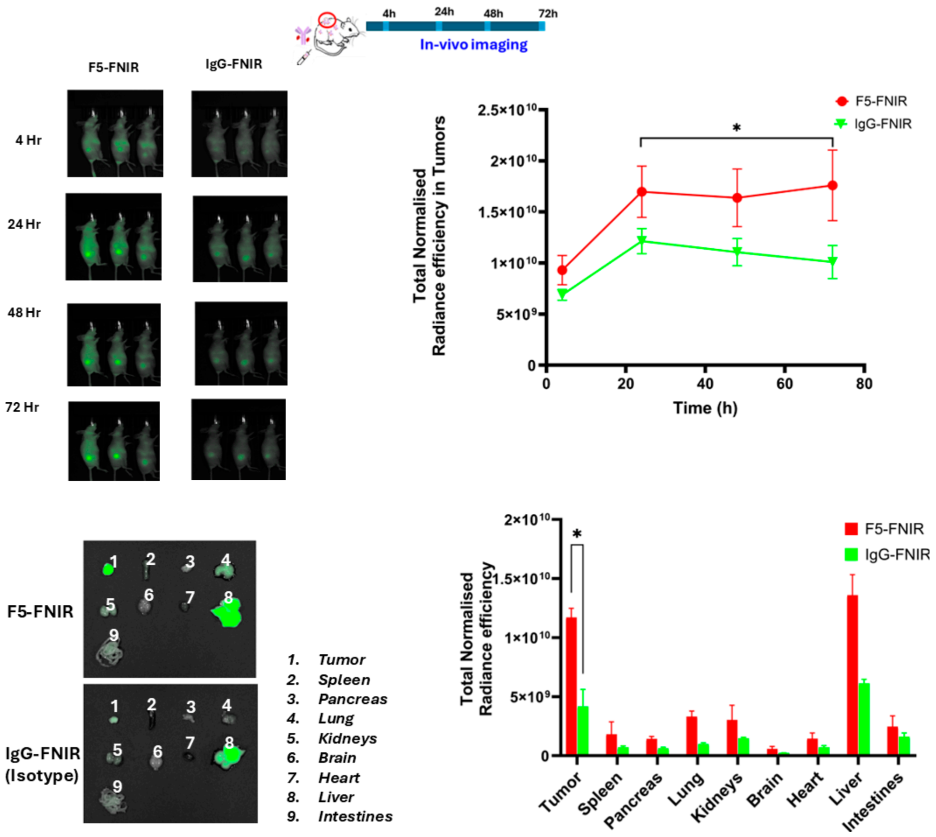

Figure S3. In-vivo (Top panes) and ex-vivo (Bottom panels) fluorescence study of F5-FNIR and Isotype Control (IgG-FNIR) in the CAPAN-1 CDX model.

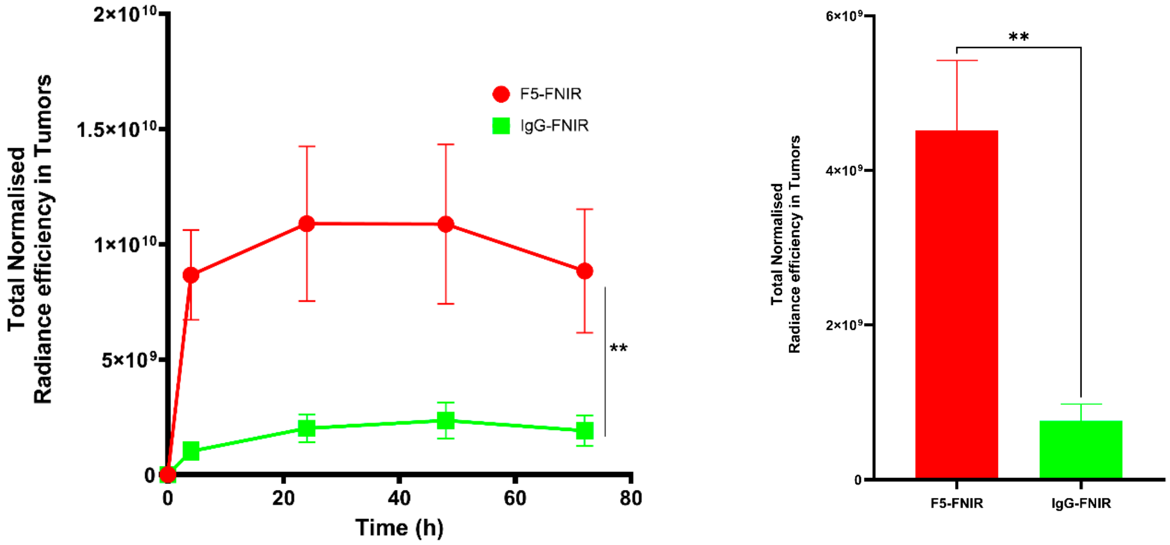

Figure S4. Biodistribution of F5-FNIR and IgG-FNIR shown over time in tumors in vivo (left panel) and ex-vivo fluorescence localization (right panel) in the BxPC3 CDX model.

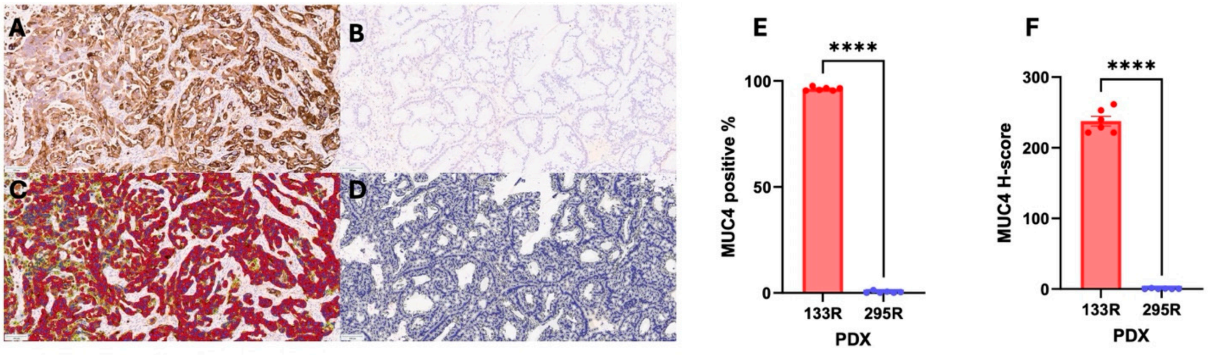

Figure S5. Quantification of MUC4 expression in PDX tumors by immunohistochemistry (IHC). Representative IHC images of MUC4 staining in tumors derived from #133R (A) and #295R (B), with corresponding pseudo-color masks illustrating staining intensity (C, D). In the pseudo-color images, nuclei are shown in blue, and MUC4 cytoplasmic staining intensity is categorized as follows: yellow = weak, orange = moderate, red = strong, and white = negative/unlabeled cells. The percentage of MUC4-positive tumor cells (E) and the MUC4 H-score (F) were quantified using HALO image analysis software. The H-score was calculated using the formula: H-score = (1 × % weakly stained cells) + (2 × % moderately stained cells) + (3 × % strongly

stained cells), resulting in a score range of 0 to 300. Statistical comparisons between tumors were performed using an unpaired t-test. ****Significant differences were observed between the two tumor sources ($p < 0.0001$).

Table S1. Binding analysis of F5 and F5-FNIR by SPR study

Sample	Koff (s^{-1})	Kon ($M^{-1}s^{-1}$)	Kd (nM)
F5-mab	1.10×10^4	1.15×10^{-4}	1.04
F5-FNIR	9.87×10^4	2.66×10^{-4}	2.70
Isotype	-	-	N.D.
FNIR dye	-	-	N.D.
